# Fatigue Behavior of Sandstone Exposed to Cyclic Point-Loading: Implications for Improving Mechanized Rock Breakage Efficiency

**DOI:** 10.3390/ma16072918

**Published:** 2023-04-06

**Authors:** Xin Cai, Jifeng Yuan, Zilong Zhou, Zhibo Wu, Jianmin Liu, Barkat Ullah, Shaofeng Wang

**Affiliations:** 1 School of Resources and Safety Engineering, Central South University, Changsha 410010, China; 2 Hunan Provincial Key Laboratory of Resources Exploitation and Hazard Control for Deep Metal Mines, Central South University, Changsha 410010, China; 3 Norinmining Co., Ltd., Beijing 100055, China; 4 CCFED Civil Engineering Co., Ltd., Changsha 410010, China

**Keywords:** cyclic point loading, rock fatigue, loading frequency, waveform, mechanized rock breakage, parameter optimization

## Abstract

During the process of mechanized excavation, rock is essentially subjected to cyclic point loading (CPL). To understand the CPL fatigue behavior of rock materials, a series of CPL tests are conducted on sandstone samples by using a self-developed vibration point-load apparatus. The effects of loading frequency and waveform on rock fatigue properties under CPL conditions are specifically investigated. The load and indentation depth histories of sandstone samples during testing are monitored and logged. The variation trends of fatigue life (failure time) under different loading conditions are obtained. Test results indicate that the fatigue life of the sandstone sample exposed to CPL is dependent on both loading frequency and waveform. As the loading frequency rises, the fatigue life of the sandstone first declines and then increases, and it becomes the lowest at 0.5 Hz. In terms of waveform, the fatigue life of the sandstone is largest under the trigonal wave and is least under the rectangular wave. These findings can provide valuable theoretical support for optimizing the rock cutting parameters to enhance the efficiency of mechanized excavation.

## 1. Introduction

Rock breakage is imperative to a variety of rock engineering projects, such as mining and tunnelling [1,2,3]. With the accelerating progress of rock engineering, the drawbacks of the drilling-and-blasting method, such as great rock damage, violent ground vibration, significant operating risk, low energy utilization, and intermittent operation, have been increasingly exposed [4,5]. These disadvantages seriously restrict the safe and efficient construction of rock engineering projects. Thus, this addresses the urgent need to revolutionize the traditional approach of rock breakage by means of explosives. Due to its superiorities such as weak disturbance to surrounding rock and continuous operation, the mechanized excavation can provide more realistic solutions to the issues induced by blasting [6].

**Figure 1:**
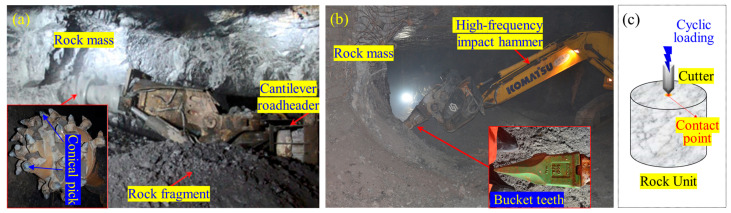
(**a**) Roadheader with pick; (**b**) high-frequency impact hammer with bucket teeth; (**c**) mechanical model of rock unit exposed to the disturbance from mechanized excavation.

In fact, the manner of non-explosive mechanized excavation has been broadly used in soft rock strata, typically in coal mines [7,8], and has been preliminarily applied in hard rock mines [9,10] or tunnels [11]. Varied rock breakage machines have also been invented, two of which are most widely employed in the mining industry, i.e., roadheader with pick and high-frequency impact hammer with bucket teeth (Figure 1a,b). Whether the roadheader or the high-frequency impact hammer is used, the contact area between the cutter and the rock is very small. Such localized contact can be considered as a contact point. Moreover, the hard rock commonly experiences multiple cuttings before fracturing because the high cutting resistance of the hard rock makes the rock breakage difficult in one cutting cycle. The mechanism of rock cutting can be simplified as the fatigue failure of rock subjected to point loading (PL), as illustrated in Figure 1c. Hence, a comprehensive and in-depth understanding of the mechanical behavior of rock exposed to cyclic point loading (CPL) is significant for non-explosive mechanized excavation.

In the past several decades, great efforts have been made to explore the rock fatigue behavior [12,13]. Numerous cyclic loading tests are performed on various rock or rock-like materials, such as salt [14,15], sandstone [16,17], dolomite [18], limestone [19,20], marble [21,22], granite [23,24,25], tuff [26,27], and concrete [28,29]. Based on these abundant publications, it is widely reported that many internal (including brittleness [19], strength [20]) and external factors (such as stress level [15,25], waveform [17], loading frequency [14,25], loading pattern [26,27], and confinement [14,15,16,22,23]) pronouncedly control the fatigue behavior of rock. Concretely speaking, Attewell and Farmer [18] pointed out that the fatigue life of limestone rises with the decreasing amplitude of cyclic load. Under a given condition of upper stress limit, loading frequency, and stress amplitude, the percentage of strain hardening is increased as the cyclic number rises. Fuenkajorn and Phueakphum [14] suggested that the number of cycles to failure is increased by the rising loading frequency but is reduced by the increasing upper stress limit. Bagde and Petroš [17] reported that under the same frequency and amplitude of cyclic loading, the square waveform results in the fastest damage accumulation in the rock sample, followed by sinusoidal and ramp waveforms. Liu and He [16] found that the confinement elevates both final deformation and the cyclic number of the accelerating phase of sandstone. These outcomes of the previous research greatly enhance the understanding of rock fatigue behavior. In the majority of previous investigations, the samples are, however, subjected to a uniform surface stress field rather than a concentrated point load [30]. The fatigue characteristics of rock materials under the CPL condition are still unknown.

This paper aims to study the fatigue behavior of sandstone exposed to cyclic point-loading. The laboratory vibration point-load apparatus that can apply CPL on rock samples is used to simulate the rock breakage process in mechanized excavation. The experimental procedures and measurement methods are also described. Several CPL tests are conducted on cylindrical sandstone samples to obtain the mechanical response of rock. The effects of the loading frequency and waveform of CPL on the fatigue behavior of the sandstone are revealed. The findings from the test results to the optimization of mechanized rock cutting are discussed.

## 2. Apparatus Design and Test Methodology

### 2.1. Apparatus Structure

**Figure 2:**
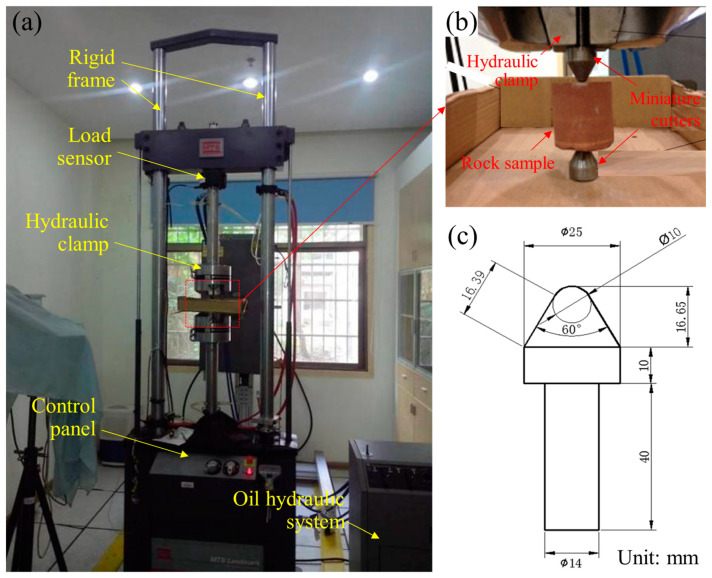
The vibration point-load apparatus: (**a**) photographic view; (**b**) detailed view of the rock sample and the miniature cutters; (**c**) geometry of the cone-shaped cutter.

The vibration point-load apparatus is placed in the advanced research center at Central South University, China. It mainly consists of a static uniaxial loading unit, a vibration excitation unit, and a point-load generating unit [30], as shown in Figure 2. The static loading unit is an electro-hydraulic servo testing machine (MTS Landmark), which has a stiffness of 467 mN/m, a maximum load of 100 kN, and a load measuring accuracy of 0.5%. The vibration excitation is controlled by an electro-hydraulic servo valve that can realize fatigue loading up to 80 Hz simultaneously with the static load. The waveforms can be adjusted by computer programs, such as triangle, rectangle, sinusoidal, and realistic complex waveforms. The loading patterns include constant-amplitude, variable-amplitude, and stochastic-amplitude classes. The point-load-generating unit is composed of the hydraulic clamp and the miniature cutter (Figure 2b). The shapes of cutters are substitutable according to the simulating rock breakage machines, such as the pick and bucket teeth. A cone-shaped cutter is designed in this study to simulate the conical pick installed in the roadheader, and its specific geometry is illustrated in Figure 2c. The cutter is made of hardened chromium alloy with 60 HRC so that they can considered as a rigid body to minimize their deformation and damage during testing. By this apparatus, we can conduct the axial CPL test on rock samples to investigate the rock breakage exposed to mechanical vibrating impact.

### 2.2. Sample Requirements

**Figure 3:**
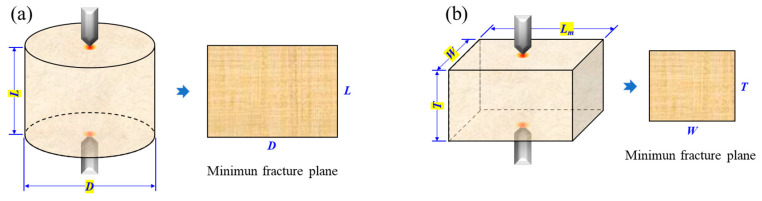
Schematics of the sample suggested in CPL testing: (**a**) cylindrical sample and (**b**) block sample (modified from [31]).

The rock samples required for CPL tests are in accordance with the standard suggested by the International Society for Rock Mechanics and Rock Engineering (ISRM) [31,32]. Both cylindrical and block samples can be used for tests, as illustrated in Figure 3. Specific requirements for sample preparation are as follows:

(1) Rock samples should be retrieved from the rock slate without visible geological weakness to minimize the property dispersion across the samples.
(2) The size of the sample should be at least 10 times greater than the average grain size in the rock.
(3) Cylindrical samples with a length/diameter (*L*/*D*) ratio of 0.3–1.0 are preferable (Figure 3a). The ends of the sample should be polished to ensure that the ends are flat to 0.02 mm and depart from perpendicularity to the axis of the sample by less than 0.001 rad.
(4) A rock block is an alternative for the sample shape (Figure 3b). The ratio of thickness (*T*) to width (*W*) should be between 0.3 and 1.0. The main side length (*L_m_*) should be at least 0.5 *W*.
(5) For routine testing, the sample should be dried (naturally dried or oven-dried) before testing to eliminate the moisture effect on the test results.

### 2.3. Testing Procedures

The CPL experiment should be executed according to the following procedures:

(1) The selected cutter is tightly installed at the hydraulic clamp.
(2) The sample is inserted between a pair of cutters that are closed to make contact along a line perpendicular to the end surfaces of the sample.
(3) The cyclic loading path is input into the computer program and then the desired load is applied on the sample until the failure of the sample.
(4) The applied load *F* and indentation depth *δ* (i.e., axial displacement) are monitored and recorded by a force sensor in the MTS landmark and a linear variable differential transformer (LVDT), respectively. The curve of *F*-*δ* of the rock sample is obtained, and the fatigue behavior of the sample can be determined accordingly. The moment when the force declines to zero is defined as the failure time of the sample.

### 2.4. Data Reduction

The point load strength (*I_s_*) of the rock sample subjected to CPL can be determined as the form in the monotonous point loading test [31]:

(1) $$I_{s}=\frac{P}{D_{c}^{2}}$$

where P is the peak load in CPL testing and $D_{c}$ is the equivalent diameter of the sample [31]:

(2) $$D_{c}^{2}=\frac{4S}{\pi }$$

where S is the minimum cross-sectional area of a plane through the cutter contacting points. As shown in Figure 3, for the cylindrical sample, S=DL; for the block sample, S=WT [31].

## 3. Experimental Schemes

### 3.1. Material Characterization and Sample Preparation

A sandstone collected from Yunnan province of China (Yunnan sandstone, YNS for short) is selected for testing in this study. The results of X-ray diffraction measurements show that the YNS sandstone is primarily composed of quartz (~54%) and feldspar (~29%) by weight. Other low-content minerals contain mica (~6%), calcite (~6%), and clay minerals (~6%). Several routine tests are conducted on the YNS samples to determine the essential physical and mechanical parameters. The YNS samples have a density of 2220 kg/m^3^ and a porosity of 7.08% measured by the nuclear magnetic resonance technique. The average uniaxial compressive strength, obtained on four standard samples, is about 45.7 MPa, and the monotonous peak point load (PL, *P_s_*) is 4.63 kN (i.e., 1.81 MPa PL strength).

All samples are drilled from a single rock slate to minimize the variation in engineering properties across the sample set. The cylindrical samples with a 50 mm diameter and 0.8 *L*/*D* ratio are manufactured. The maximum grain size of YNS is about 0.5 mm, which is far less than the sample size. The other requirements in Section 2.2. are strictly satisfied. All prepared samples are placed in a well-ventilated laboratory for air-drying over seven days and are then sealed with a plastic wrap to prevent moisture in the atmosphere before testing [33,34].

### 3.2. Loading Schemes

In the present study, two groups of tests are designed to investigate the influences of loading frequency and waveform on rock fatigue behavior under CPL conditions. The specific loading schemes are introduced in the following subsections.

#### 3.2.1. CPL Testing with Different Loading Frequencies

**Figure 4:**
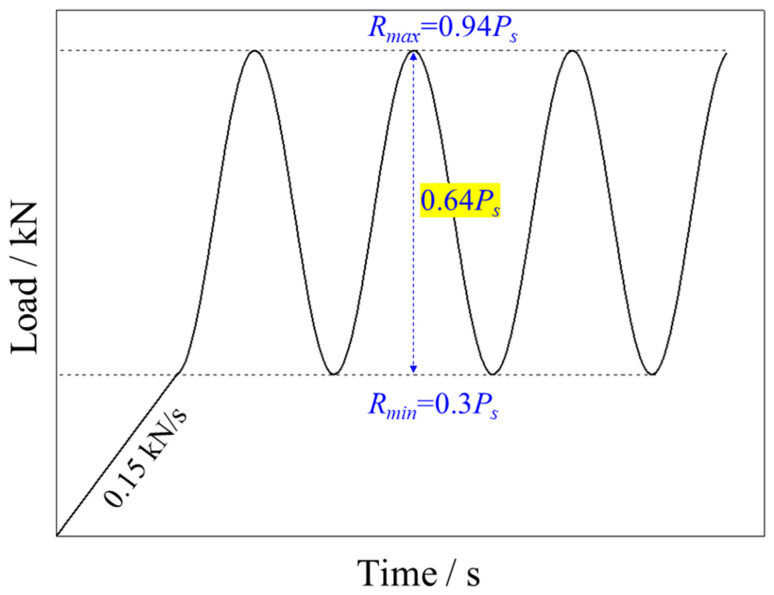
Schematic of force history in CPL tests.

In the first set of experiments, to study the effects of loading frequency on rock fatigue behavior subjected to CPL, the loading frequency is set as the single independent variable between the testing groups. As shown in Figure 4, the sinusoidal waveform is selected with the constant lower (*R_min_*)- and upper (*R_max_*)-load limits of 1.39 kN (~0.3 *P_s_*) and 4.35 kN (~0.94 *P_s_*) respectively, such that the amplitude of the wave is sustained at 0.64 *P_s_*. The range of loading frequency is between 0.1 and 5 Hz.

The force control mode is chosen for loading in all tests. The static load applied on the rock sample is first increased from 0 to 1.39 kN at a constant loading rate of 0.15 kN/s. Subsequently, the static load is sustained, with which an axial CPL with 2.96 kN amplitude and various frequencies is superimposed until the failure of the sample. The loading schemes and test results are listed in Table 1.

#### 3.2.2. CPL Testing with Different Waveforms

In the second set of experiments, the waveform is the single independent variable. Three waveforms, i.e., trigonal, sinusoidal, and rectangular waves, are used for tests with a constant loading frequency of 0.5 Hz. In addition, the lower and upper load limits are 1.39 kN and 4.35 kN, respectively, and are consistent in all tests.

Similar to the first set in Section 3.2, the load is exerted through the force control mode. First, the static load is applied to 1.39 kN by a rate of 0.15 kN/s. The CPLs with different waveforms are superimposed with the static load until the rock failure. The loading parameters and experimental results are summarized in Table 2.

## 4. Experimental Results and Discussion

### 4.1. Effects of Loading Frequency on Rock Behavior under Cyclic Point Loading

#### 4.1.1. Failure Pattern

**Figure 5:**
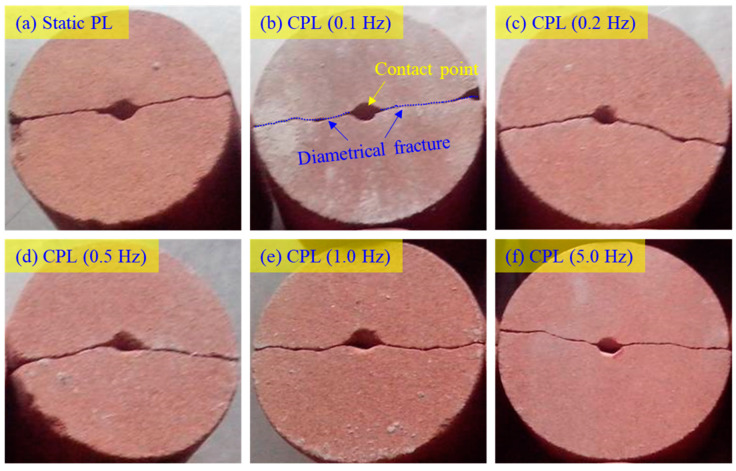
Failure pattern of YNS subjected to (**a**) monotonous point loading and (**b**–**f**) CPLs with different frequencies.

Figure 5 illustrates the failure patterns of YNS samples in monotonous and cyclic point loading tests. Regardless of the loading condition, the failure patterns are very similar. A couple of spherical indentations is clearly observed on the center of the sample surfaces. A diametrical fracture surface splits the sample into halves, which agrees with the failure pattern of Gosford Sandstone subjected to monotonous point loading [35]. This diametrical fracturing should be attributed to the tensile failure induced by point indentation.

#### 4.1.2. Load-Indentation Depth Curves and Fatigue Life of Sandstone

**Figure 6:**
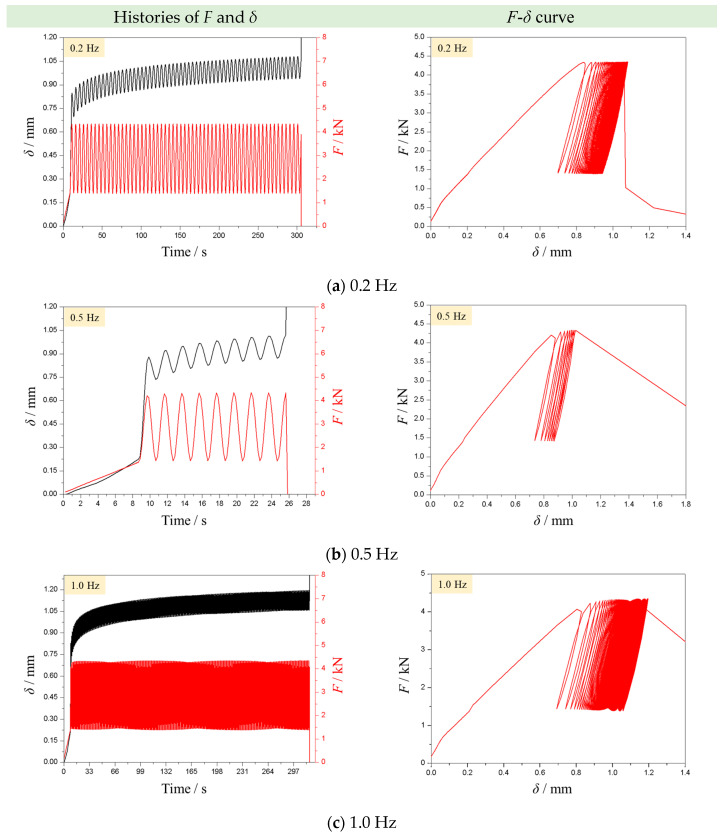
Typical curves of load and indentation depth in CPL tests with the frequencies of (**a**) 0.2 Hz, (**b**) 0.5 Hz, and (**c**) 1.0 Hz (the left column shows the histories of *F* and *δ*, and the right column shows the *F*-*δ* curves).

Figure 6 presents the typical histories of force (*F*) and indentation depth (*δ*) of samples in CPL tests with the loading frequencies of 0.2, 0.5, and 1.0 Hz. The lower and upper load limits, as well as loading frequencies, are precisely imposed on the rock sample according to our settings. The wave amplitude is constant before the rock failure. In each loading cycle, the indentation depth is changed synchronously with the load. From the view of the whole loading process, the lower and upper values of the indentation depth gradually rise with increasing cyclic number. The hysteresis loops are clearly observed in the *F-δ* curve. The density of the hysteresis loop apparently increases as the cyclic number rises until the failure of the rock sample. Once the rock sample fails, the load drops abruptly while the indentation depth surges sharply. All observations indicate that the proposed apparatus can implement the CPL test to obtain the point-loading fatigue behavior of the rock sample.

**Figure 7:**
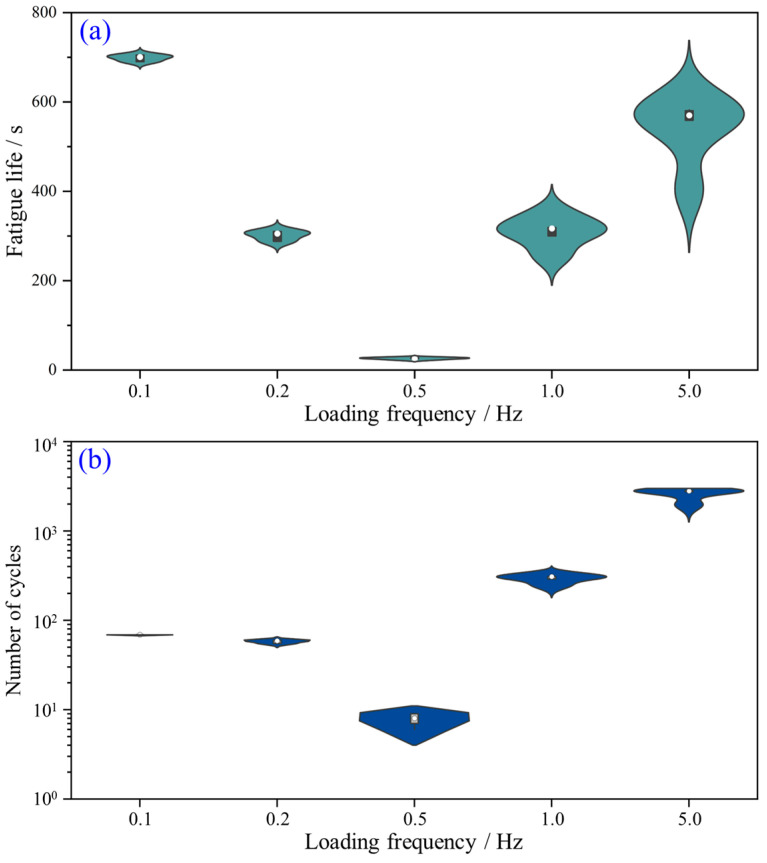
Variations in (**a**) fatigue life and (**b**) number of cycles of YNS sample against loading frequency under CPL condition.

The fatigue life is an important indicator for evaluating the efficiency of rock breakage. In this study, we regard the time duration from the loading initiation to the total failure of rock as the fatigue life of the sample. From Figure 6, it can be seen that under various loading frequencies, the experienced cycle numbers of the rock sample are much different. This means that the loading frequency plays the role of a controlling factor in the fatigue life of the rock sample. Figure 7a further gives the variation in the fatigue life of the YNS samples against the loading frequency. Interestingly, with the increase in loading frequency, the fatigue life of rock declines first and then rises. The value of the fatigue life becomes the least at the loading frequency of 0.5 Hz. The number of cycles changes against the loading frequency in a similar manner of fatigue life (Figure 7b).

It is shared that the fatigue life of rock mainly depends on the correlation between the loading rate and the speed of crack growth [12]. If the loading rate is high enough (high loading frequency), the rapid increase in load limits the development and nucleation of cracks or flaws, and further raises the fatigue life of rock. On the contrary, when the loading rate is very low, the majority of cracks developed in the cycle are closed again before the beginning of the next loading cycle. Hence, we can reasonably speculate that rock material has a minimum fatigue life at a specific loading frequency. In other words, there is an optimal loading frequency for rock breakage. The optimal rock breaking frequency for the tested YNS sample is nearly 0.5 Hz under the fixed upper and lower load limits.

It is worth noting that the observations in Figure 7 disagree with the prior study [25], in which the number of cycles of Alvand monzogranite monotonously rises at higher loading frequencies (from 0.1 to 5 Hz) under cyclic uniaxial compression. One possible reason is that the optimal loading frequencies for rock failure are different for various rock types. The other interpretation is that the alteration in loading type (point loading or surface loading) changes the optimal loading frequency.

### 4.2. Effects of Waveform on Rock Behavior under Cyclic Point Loading

#### 4.2.1. Failure Pattern on Rock Behavior under Cyclic Point Loading

**Figure 8:**
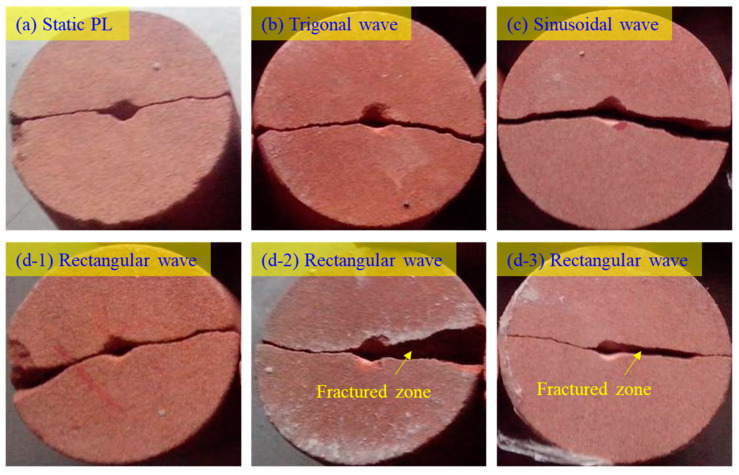
Failure patterns of the YNS sample subjected to (**a**) static point loading and (**b**–**d**) CPLs with different waveforms.

The failure patterns of the YNS samples subjected to CPL with trigonal and sinusoidal waves are similar to those in monotonous PL tests, as shown in Figure 8. An interesting observation is that under the CPL with rectangular waves, the fractured zones occur near the contact point in the majority of samples. It is hypothesized that the steep front of the rectangular wave leads to a larger damage area on the end surface of the sample. Contrarily, both the trigonal and sinusoidal waves have a slower rising front such that the force is more concentrated on the contact point. The damage is totally generated on the contact point rather than on the surface near the contact point.

#### 4.2.2. Load-Indentation Depth Curves and Fatigue Life of Sandstone

**Figure 9:**
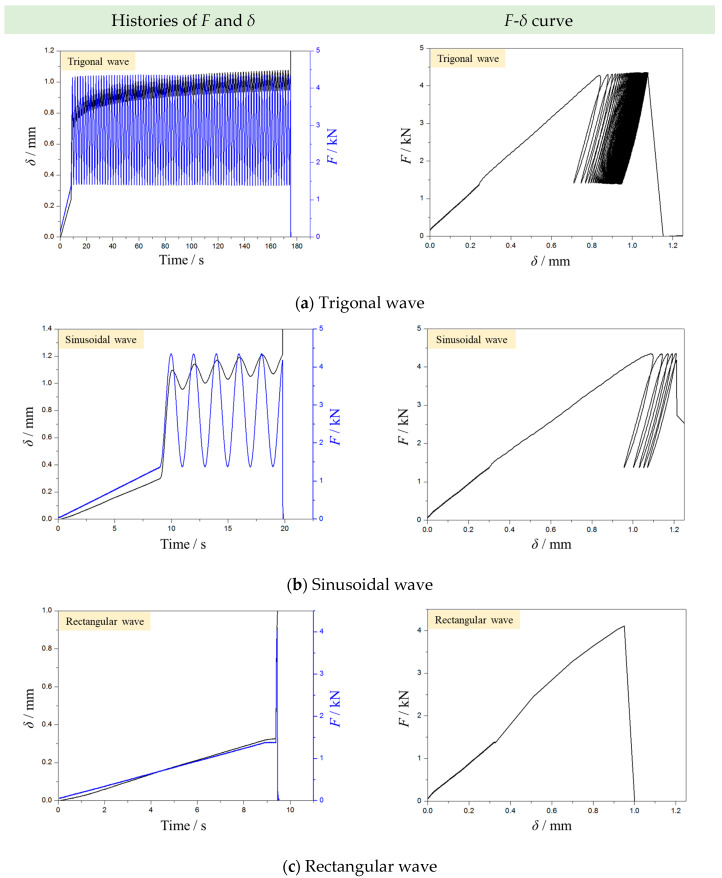
Typical curves of load and indentation depth in CPL tests with (**a**) trigonal, (**b**) sinusoidal, and (**c**) rectangular waves (the left column shows the histories of *F* and *δ*, and the right column shows the *F*-*δ* curves).

Figure 9 displays the typical histories of *F* and *δ* of samples in CPL tests with three different waveforms. The waveform significantly affects the CPL fatigue behavior of the rock sample. For the trigonal wave (Figure 9a), the YNS sample fails after more than 80 point-loading cycles. When the sinusoidal wave is applied, the sample only experiences much fewer cycles (5–12 cycles). The point load with the rectangular wave destroys the sample in the first cycle. This means that the rectangular wave can notably promote the rock breakage.

**Figure 10:**
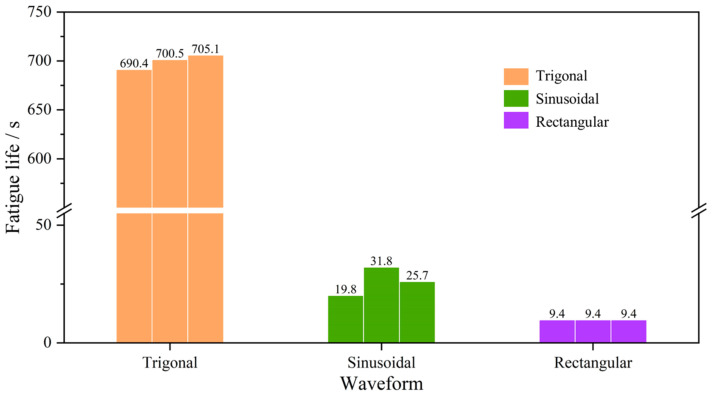
CPL fatigue lives of the YNS samples subjected different waveforms.

To understand the effect of waveform on the CPL fatigue life of rock, the histogram of the CPL fatigue life of each YNS sample exposed to trigonal, sinusoidal, and rectangular waves is plotted in Figure 10. It can be clearly observed that under the trigonal wave, the YNS sample has the average CPL fatigue life of 698.7 s, which is the maximum in the three tested waves. The CPL fatigue life of the sample subjected to the sinusoidal wave is 77.3 s, which is reduced by 88.9% compared to the life under the trigonal wave. The fatigue life of the sample under the rectangular wave is the least, which is less than 10 s. According to previous studies [17,36], the shape of the waveform plays a predominant role in the fatigue life of rock. At a given loading frequency and amplitude, the rectangular waveform is the most severe testing condition and leads to the shortest fatigue life of rock due to its high loading rate, a great change in loading rate, and long residence period. The trigonal waveform has the slowest damage accumulation probably because of its constant and lowest loading rate. From the view of mechanized excavation, this phenomenon indicates that the rectangular wave has the highest efficiency of rock breakage. This provides a valuable hint that the rectangular wave should be prioritized for the rock breakage machine.

## 5. Conclusions and Prospects

In this study, a self-developed vibration point-load apparatus determining the fatigue behavior of rock materials under cyclic point loading (CPL) is introduced. By this device, two sets of CPL tests are performed on cylindrical YNS samples. The influences of loading frequency and waveform on the fatigue responses of the YNS samples are preliminarily investigated. The main conclusion of this study can be drawn as follows:

(1) The fatigue behavior of rock under CPL conditions is greatly dependent on loading frequency. The fatigue life of the YNS sample shows a trend of “decline followed by rise” with the increase in loading frequency. The minimum value of the YNS sample is 0.5 Hz.
(2) The waveform also plays a controlling role in the fatigue behavior. The order of the fatigue life from largest to least is as follows: trigonal wave > sinusoidal wave > rectangular wave. In addition, when subjected to rectangular waveforms, the fractured zone can be observed on the rock surface. The rectangular waveform has the most severe damage on rock among the three tested waves.
(3) The enlightenment of this work to mechanized excavation is that the efficiency of rock breakage (i.e., fatigue life) is significantly controlled by the parameters of the rock breaking machine, such as loading frequency and waveform. For a given rock type, there is an optimal combination of rock cutting parameters probably including loading frequency, waveform, amplitude, and upper and lower load limits. In rock engineering practice, similar CPL tests should be first conducted on the rock sample gathered from the site to predetermine the optimal combination of rock cutting parameters. Then, the optimal parameters should be applied to the mechanized machine, such that the efficiency of rock breakage can be markedly improved.

This study is still limited to one rock type (Yunnan sandstone) and two loading parameters (loading frequency and waveform). In future work, the impacts of other loading parameters such as cutter shape, amplitude, and upper and lower load limits should be explored intensively. Moreover, the CPL tests will be further performed on other types of hard rocks, such as limestone, marble, and granite, to testify whether our findings are more generally applicable.

## Figures and Tables

**Table 1:** Test parameters and results in CPL testing with different loading frequencies.

Sample No.	Upper Limit (kN)	Lower Limit (kN)	*f* (Hz)	Number of Cycles	Fatigue Life ^a^ (s)
D-1-1	0.94 *P_s_*	0.3 *P_s_*	0.1	68	690.4
D-1-2				69	700.5
D-1-3				69	705.1
D-2-1	0.94 *P_s_*	0.3 *P_s_*	0.2	60	310.1
D-2-2				55	288.2
D-2-3				59	305.3
D-3-1	0.94 *P_s_*	0.3 *P_s_*	0.5	8	25.6
D-3-2				9	28.3
D-3-3				6	22.5
D-3-4				7	25.6
D-3-5				9	28.1
D-4-1	0.94 *P_s_*	0.3 *P_s_*	1.0	244	255.1
D-4-2				309	316.7
D-4-3				290	300.4
D-4-4				339	350.1
D-4-5				308	319.1
D-5-1	0.94 *P_s_*	0.3 *P_s_*	5	2846	580.3
D-5-2				1961	403.1
D-5-3				2741	559.1
D-5-4				2856	582.1
D-5-5				2796	570.1

^a^ The time duration from the loading initiation to rock failure is defined as the fatigue life of the sample.

**Table 2:** Test parameters and results in CPL testing with different waveforms.

Sample No.	Waveform	Upper Limit (kN)	Lower Limit (kN)	*f* (Hz)	Number of Cycles	Fatigue Life ^b^ (s)
T-1	Trigonal	0.94 *P_s_*	0.3 *P_s_*	0.5	84	690.4
T-2					80	700.5
T-3					90	705.1
S-1	Sinusoidal	0.94 *P_s_*	0.3 *P_s_*	0.5	5	19.8
S-2					12	31.8
S-3					8	25.7
R-1	Rectangular	0.94 *P_s_*	0.3 *P_s_*	0.5	<1 ^a^	9.4
R-2					<1 ^a^	9.4
R-3					<1 ^a^	9.4

^a^ The samples fail in the first cycle of point loading. ^b^ The time duration from the loading initiation to rock failure is defined as the fatigue life of the sample.
